# Prevalence of negative life events and chronic adversities in European pre- and primary-school children: results from the IDEFICS study

**DOI:** 10.1186/0778-7367-70-26

**Published:** 2012-11-22

**Authors:** Barbara Vanaelst, Inge Huybrechts, Ilse De Bourdeaudhuij, Karin Bammann, Charalambos Hadjigeorgiou, Gabriele Eiben, Kenn Konstabel, Nathalie Michels, Denes Molnar, Luis A Moreno, Iris Pigeot, Lucia Reisch, Alfonso Siani, Krishna Vyncke, Stefaan De Henauw

**Affiliations:** 1 Department of Public Health, Ghent University, UZ-2BlokA De Pintelaan 185, 9000, Ghent, Belgium; 2 Research Foundation – Flanders (FWO), Egmontstraat 5, Brussels, Belgium; 3 Dietary Exposure Assessment Group, International Agency for Research on Cancer (IARC), Lyon, France; 4 Department of Movement and Sport Sciences, Ghent University, Watersportlaan 2, 9000, Ghent, Belgium; 5 Bremen Institute for Prevention Research and Social Medicine, University of Bremen, Achterstr. 30, 28359, Bremen, Germany; 6 Institute for Public Health and Nursing Care Research, University of Bremen, Postfach 330440, 28344, Bremen, Germany; 7 Research & Education Institute of Child Health, 8 Attikis Str, 2027, Strovolos, Cyprus; 8 Department of Public Health and Community Medicine, Public Health Epidemiology Unit, Sahlgrenska Academy, University of Gothenburg, Gothenburg, Sweden; 9 National Institute for Health Development, Hiiu 42, 11619, Tallinn, Estonia; 10 National Institute of Health Promotion, University of Pécs, Gyermekklinika, József Attila utca 7, 7623, Pécs, Hungary; 11 GENUD (Growth, Exercise, Nutrition and Development) research group, School of Health Sciences, University of Zaragoza, Domingo Miral s/n, 50.009, Zaragoza, Spain; 12 Department of intercultural communication and management, Copenhagen Business School, Porcelanshaven 18A, DK-2000, Frederiksberg, Denmark; 13 Epidemiology & Population Genetics, Institute of Food Sciences, CNR, Via Roma 64, 83100, Avellino, Italy

**Keywords:** Child, Life events, Adversity, Prevalence, Stress, Epidemiology

## Abstract

**Background:**

Children are not always recognized as being susceptible to stress, although childhood stressors may originate from multiple events in their everyday surroundings with negative effects on children’s health.

**Methods:**

As there is a lack of large-scale, European prevalence data on childhood adversities, this study presents the prevalence of (1) negative life events and (2) familial and social adversities in 4637 European pre- and primary-school children (4–11 years old), using a parentally-reported questionnaire embedded in the IDEFICS project (‘Identification and prevention of Dietary- and lifestyle-induced health EFfects In Children and infantS’).

**Results:**

The following findings were observed: (1) Certain adversities occur only rarely, while others are very regular (i.e. parental divorce); (2) A large percentage of children is shielded from stressors, while a small group of children is exposed to multiple, accumulating adversities; (3) The prevalence of childhood adversity is influenced by geographical location (e.g. north versus south), age group and sex; (4) Childhood adversities are associated and co-occur, resulting in potential cumulative childhood stress.

**Conclusions:**

This study demonstrated the importance of not only studying traumatic events but also of focusing on the early familial and social environment in childhood stress research and indicated the importance of recording or monitoring childhood adversities.

## Background

For a long time, stress has incorrectly been assumed to predominantly manifest in adults. Many investigators have however recently turned to the incidence of stress in children [1-10]. Sandberg defined childhood stress as ‘any intrusion into the children’s normal physical or psychosocial life experiences that acutely or chronically unbalances their physiological or psychological equilibrium, threatens security or safety, or distorts their physical or psychological growth or development’ [11]. In this definition, three stress components can be distinguished: 1) the environmental sources of stress or so-called ‘stressors’ (e.g. negative life events or more chronic adversities in the children’s school-, family- or inter-personal environment), 2) the psychological response given to these stressors (e.g. emotions), and 3) the biological stress response provoked by stressor exposure (e.g. the hormonal stress response) [12,13]. This paper focusses on childhood stressor exposure; more specifically on the occurrence of negative life events and adversities of familial and social nature.

In particular chronic exposure to adverse, stressful situations may affect children’s behaviour and personality development and may have consequences on both their physiological and psychological health, with effects potentially persisting into adolescence and adulthood (e.g. depression, affective disorders, cardiovascular or auto-immune diseases, psychosomatic complaints, substance abuse) [14-20]. While some children may be relatively shielded from adversities, others may be exposed to a multiplicity of successive hardships or life-course-transitions resulting in cumulative stress [21].

The most obvious demographic change in Western Europe are the increased divorce rates, which may impact on the children’s everyday life through, e.g., a changing family structure [22]. As the family environment may affect the social, emotional and physical health of children, it should be considered an important factor in the child’s well-being [23,24]. Moreover, stressors from familial origin may not be isolated events, but cluster together or give rise to other unfavourable events (e.g. parental divorce may lead to organizational changes, decreased economic resources and parental strains), all together highlighting the importance of considering the early family and social environment in childhood stress research.

Despite the importance of recording/monitoring childhood adversities, there is a lack of large-scale, international research on the prevalence of negative life events and familial and social conditions which may constitute potential childhood adversity. Moreover, the majority of previous stress research has focused on rare traumatic events without considering familial and social conditions. Therefore, this study examines the prevalence of (1) negative life events (NLE) and (2) familial and social adversities (FSA) in a large population of European pre- and primary-school children (4–11 years old) cross-nationally, by investigating the following research questions: (1) Is the prevalence of adversity in pre- and primary-school children equally distributed over region, age and sex group [25,26] ? (2) Can co-occurence and associations between adversities be demonstrated in this young childhood population (e.g. do certain adversities lead to other adversities or tend to co-occur) ?

## Methods

### Participants

Information on NLEs and FSAs in the child’s life was parentally reported for 4637 children (aged 4 to 11.8 years, mean (M)=7.91, standard deviation (SD)=1.80, 49.5% boys). This was part of the follow-up survey (September 2009 - May 2010) of the IDEFICS study, an Integrated Project within the 6^th ^Framework Programme of the European Commission (‘Identification and prevention of Dietary- and lifestyle-induced health EFfects In Children and infantS’, http://www.idefics.eu).

The IDEFICS project is a multicentre longitudinal intervention study of pre- and primary-school children in 8 European countries (Belgium, Cyprus, Estonia, Germany, Hungary, Italy, Spain, Sweden), investigating the aetiology of diet- and lifestyle-related diseases and disorders in children. In this project, also community-oriented prevention programmes for obesity are developed (working on the level of diet, physical activity and stress reduction) and evaluated in a controlled study design [27]. In each country, one intervention and one control region was selected which were comparable with regard to infrastructural, socio-demographic and socio-economic characteristics. All children residing in the selected intervention and control regions who were within the defined age group of 2–9 years old at baseline, were eligible for participation to IDEFICS. Because of budgetary constraints and feasibility considerations, it was not intended to generate a representative sample of a given country or Europe in general.

**Figure 1:**
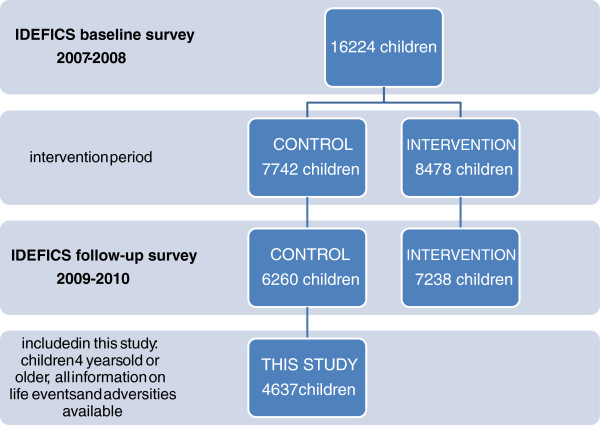
Study flow-chart presenting participation information of the baseline and follow-up survey of the IDEFICS project, and the total number of children included in the presented analysis.

The baseline survey started in 2007 with a cohort of 16224 children which were approached through school and kindergarten settings using a letter and leaflet addressed to the parents (Figure 1). The follow-up survey resulted in a total sample size of 13498 children. More detailed research goals, methodology and instruments of IDEFICS have been described elsewhere [28].

As one of the IDEFICS intervention modules was directed at stress and stress-coping capacity on community-, school- and family-level [27], we decided to only include the control regions of the participating countries in this study to rule out intervention-bias on the studied variables (N=6260/13504; 46.4%). Statements in this study regarding regional variations thus only relate to the participating control regions and should not be considered as representative for the respective countries. Children younger than 4 years of age (N=69) and children from whom any adversity information was missing were excluded from the analysis (N=1623/6260; 25.9%). This resulted in a final sample size of 4637 participants, which is schematically presented in Figure 1. No differences were found between the included and excluded group for sex and age, while low parental education was more prevalent in the excluded group (data not shown). The study was conducted according to the guidelines of the Declaration of Helsinki and approvals of the local Ethical Committees were obtained for each survey centre.

### Childhood adversities

Life events are generally assumed to represent a basis for experiencing stress as they are accompanied by undesirable demands and threats and lead to changes in a person’s life. Therefore, questionnaires assessing life events and adversities are considered estimates of stress exposure [12,29]. In this study, childhood adversity was studied using a parent-reported questionnaire on adversity and life events, i.e. the ‘IDEFICS Parental Questionnaire’, including information on socio-demographics, family lifestyle, life events and wellbeing of the children. The quality of the questionnaire and comparability across the survey centres was assured by a translation/back-translation procedure for each local language and by re-administering the parental questionnaire to a convenience sample of study participants [28,30].

**Figure 2:**
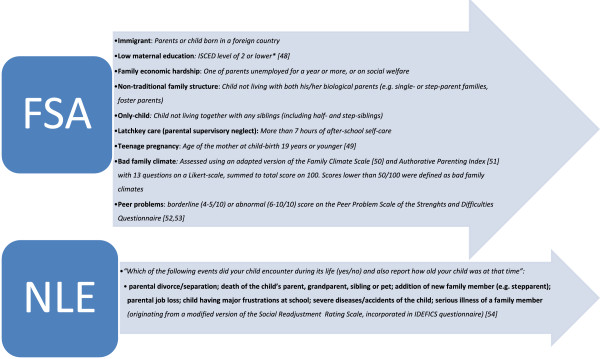
**Overview of Familial and Social Adversities (FSA) and Negative Life Events (NLE) variables as assessed in the IDEFICS project (2009–2010) **[48-54].

Parental conflicts or divorce [31], low supportive or unfavourable family climates [32-35], domestic violence or abuse [36], parental supervisory neglect [37,38], socio-economic disadvantage [39-42], serious illness of the child or a family member [43,44], death of a child’s parent, grandparent, sibling or pet [19], and peer problems or frustrations at school [45-47] have in literature all been shown to emotionally and psychologically affect children. Therefore, parents were asked to complete questions on both the life-time occurrence of the above-mentioned negative life events (NLE) and the more chronic familial and social situations which may constitute potential childhood adversity (FSA: familial and social adversities), such as ethnicity of the family, education of the mother, employment of the parents, family structure and family relationships. These childhood adversity variables were all of dichotomous nature (occurrence or no occurrence of the event; presence or no presence of the adversity). Figure 2 presents an overview of the studied FSA and NLE variables, their assessment and reference to literature. To accurately report on maternal education, family economic hardship and family climate, only data provided by biological-, adoptive-, or stepparents was included. For the other variables also reporting by foster-parents or family members was allowed.

Important to note is that the authors do not consider these variables as actual childhood stressors but rather as potential stressful conditions during childhood.

### Statistical analysis

Statistical analyses were performed using PASW Statistical Program version 18.0.0 (SPSS Inc, IBM, IL, USA). Each year of age was considered as one age group except children of 10 and 11 years old who were taken together in the age group ‘10’ because of the low number of 11 year olds (N=40). Regional differences were studied by grouping the countries along a north (Sweden, Estonia) - east (Hungary) - south (Italy, Spain, Cyprus) - west cluster (Belgium, Germany), based on the geographical grouping of countries by the United Nations Statistics Division [55]. Cumulative stress from FSAs and NLEs was studied by summing the number of FSAs and NLEs [3,8,56-59]. To study regional variations and differences among age groups and sexes in the prevalence of FSAs and NLEs, Pearson χ^2 ^analysis were performed. One-way ANOVA analyses were performed to study continuous variables between groups. Odds ratios (OR) and 95% confidence intervals (CI) were used to report on the co-occurrence of adversities, and the risk (or likelihood) for being exposed to a certain adversity, given another adversity was already present. As we did not aim to determine the unique contribution of each FSA/NLE adjusted for other FSAs/NLEs, unadjusted, univariate (and not multivariate) OR’s were presented (which are suitable to demonstrate the associations and co-occurrence between adversities). To correct for multiple testing a Bonferroni correction was applied: p-values <0.002 were considered statistically significant for all tests. P-values between 0.002 and 0.05 were denoted as borderline significant.

## Results

### Prevalence of FSAs and NLEs

Table 1 presents the prevalence of FSAs and NLEs for each survey centre separately and for grouped countries: the three most prevalent FSAs/NLEs are marked numerically and for each FSA/NLE the survey centre or country group with the highest prevalence is indicated in bold. A non-traditional family structure, being only-child or immigrant are the three most reported FSAs overall, while parental divorce/separation, addition of a new family member and parental job loss are the most reported NLEs.

**Table 1:** Prevalence of chronic adversities and negative life events in pre- and primary-school children participating in the IDEFICS study (2009–2010): survey centre and regional variations

		**Survey centres**									**Country groups**				
	**Total**	**Italy**	**Estonia**	**Cyprus**	**Belgium**	**Sweden**	**Germany**	**Hungary**	**Spain**	**P (χ**^**2**^**)**^**a**^	**North**^**c**^	**Easth**^**d**^	**South**^**e**^	**West**^**f**^	**P (χ**^**2**^**)**^**a**^
**Number of children included (N)**	4637	560	787	601	605	543	376	666	499		1330	666	1660	981	
**Familial and social adversities**	*Prevalence % (N)*	*Prevalence %*													
Being immigrant	③ **13.0 (604)**	19.6	6.5	**33.1**	2.8	15.8	23.1	2.9	7.0	<0.001	10.3	2.9	**20.7**	10.6	<0.001
Low maternal education	11.3 (524)	28.9	3.4	3.0	2.5	4.4	**51.9**	2.9	12.8	<0.001	3.8	2.9	14.7	**21.4**	<0.001
Family economic hardship	4.1 (191)	7.1	2.7	2.2	1.2	0.9	**9.3**	5.7	6.4	<0.001	2.0	**5.7**	5.1	4.3	<0.001
Non-traditional family structure	① **21.0 (973)**	**30.2**	24.1	28.8	15.4	13.3	22.6	24.2	6.0	<0.001	19.7	**24.2**	22.4	18.1	0.007
Single-parent family	13.1 (608)	**25.5**	12.8	23.6	5.0	4.1	14.6	13.2	5.4	<0.001	9.2	13.2	**18.8**	8.7	<0.001
Stepparent family	4.1 (192)	0.4	**8.0**	1.2	5.3	3.5	7.4	6.0	0.2	<0.001	**6.2**	6.0	0.6	6.1	<0.001
Only-children	② **16.4 (759)**	14.1	**27.2**	8.7	8.3	9.2	24.2	20.7	17.0	<0.001	19.8	**20.7**	13.0	14.4	<0.001
Latchkey care	5.6 (261)	1.3	**22.5**	3.3	0.3	7.6	0.8	1.2	0.6	<0.001	**16.4**	1.2	1.8	0.5	<0.001
Bad family climate	1.3 (58)	1.3	2.3	1.3	0.5	0.0	**2.4**	0.3	2.2	<0.001	1.4	0.3	1.6	1.2	0.097
Teenage pregnancy	2.2 (101)	2.3	**6.1**	3.0	0.5	0.7	2.4	0.6	0.4	<0.001	**3.9**	0.6	2.0	1.2	<0.001
Peer problems	8.5 (395)	9.3	7.6	**11.5**	7.8	4.6	9.8	11.0	6.4	<0.001	6.4	**11.0**	9.2	8.6	0.003
**Negative life events**	*Prevalence % (N)*	*Prevalence %*													
Parental divorce/separation	① **13.0 (602)**	3.9	**23.0**	7.3	14.7	10.7	18.6	17.4	4.4	<0.001	**18.0**	17.4	5.3	16.2	<0.001
Addition of a new family member	② **12.4 (573)**	3.4	14.0	4.5	13.2	**29.8**	13.0	9.5	12.6	<0.001	**20.5**	9.5	6.6	13.1	<0.001
Parental job loss	③ **8.7 (403)**	3.2	**14.5**	4.3	4.0	8.8	6.6	13.1	12.2	<0.001	12.2	**13.1**	6.3	5.0	<0.001
Severe diseases/accidents of the child	7.2 (333)	5.4	11.3	4.3	4.5	3.9	6.9	**13.4**	5.0	<0.001	8.3	**13.4**	4.9	5.4	<0.001
Serious illness of a family member	1.9 (90)	2.3	1.0	0.8	**4.5**	3.1	2.7	1.1	0.6	<0.001	1.9	1.1	1.3	**3.8**	<0.001
Major frustration at school	7.4 (344)	5.9	7.5	4.7	8.3	7.0	**11.4**	10.2	5.0	<0.001	7.3	**10.2**	5.2	9.5	<0.001
Death of a parent	0.7 (33)	0.7	0.5	1.0	0.7	0.4	0.8	1.4	0.2	0.339^b^	0.5	1.4	0.7	0.7	0.177^b^
Death of a sibling	0.6 (26)	0.2	0.6	0.3	1.2	0.4	1.1	0.6	0.2	0.253^b^	0.5	0.6	0.2	1.1	0.034^b^
Death of a grandparent	4.8 (221)	6.4	3.6	2.0	5.1	5.2	**7.4**	3.8	6.6	<0.001	4.2	3.8	4.9	6.0	0.121
Death of a pet	0.7 (32)	0.0	1.8	0.0	1.0	0.0	**2.4**	0.2	0.4	<0.001	1.1	0.2	0.1	**1.5**	<0.001^b^

^a^ Pearson χ^2 ^test to compare frequencies across countries, ^b^ Fischer's exact test to compare frequencies between countries, ^c^ Sweden-Estonia, ^d^ Hungary, ^e^ Italy-Cyprus-Spain, ^f^ Belgium-Germany.

①②③ Top three of most prevalent FSAs/NLEs in total are marked.

For each adversity or event, the survey centre or country group with the highest prevalence is indicated in bold if statistically different from the other survey centres or country groups.

#### Influence of region of the prevalence of FSAs and NLEs

Adversity percentages differ significantly between survey centres and country groups. Table 1 shows the highest prevalence rates of parental divorce/separation, addition of new family member, stepparent families, teenage pregnancy and latchkey care in the north; being immigrant and single-parent families appear most in the south; low maternal education, illness of a family member and death of a pet in the west; while in the east the following adversities peak: family economic hardship, non-traditional family structure, being only-child, parental job loss, severe diseases/accidents of the child, and peer problems and major frustrations at school. Although family economic hardship has the highest prevalence in the east, it should be marked that this prevalence is comparable to the south percentage. The same is true for the prevalence of only-children which occurs quite equally in the north and the east, and for the prevalence of stepparent families which occurs equal in north, east and west. In summary, Table 1 demonstrates large regional variations particularly in family structure: the prevalence of parental divorce/separation and stepparent families is high and comparable for the north, east and west, while being low in the south; single-parent families occur significantly more in the south.

#### Influence of sex on the prevalence of FSAs and NLEs

For boys and girls, no significant differences in FSAs and NLEs are observed, except for severe diseases/accidents of the child (which is more prevalent in boys (8.3% boys, 6.1% girls, p=0.004)). When examined for all age groups separately, peer problems are more prevalent in boys, more specifically in the group of 9 year olds (12.1% boys, 7.8% girls, p=0.015) (data not shown).

#### Influence of age on the prevalence of FSAs and NLEs

Childhood adversities are more prevalent in older age groups. Significant increases in the prevalence over the age groups are found for low maternal education (p < 0.001, ranging from 6.2% to 17.1% over the age groups), non-traditional family structure (p < 0.001, ranging from 14.7% to 25.8%), latchkey care (p < 0.001, ranging from 0% to 12.7%), parental divorce/separation (p < 0.001, ranging from 10% to 19.2%), major frustrations at school (p < 0.001, ranging from 3.9% to 10.4%) and peer problems (p=0.037, ranging from 5.8% to 10.0%) (data not shown).

### Cumulative stress from FSAs and NLEs

Table 2 demonstrates that 46.6% and 59.7% of the children have not yet experienced any of the studied FSAs or NLEs respectively, or that (vice versa) 53.4% and 40.3% of the children experienced at least one FSA or NLE. With an increasing sum of FSAs or NLEs the percentage of children decreases. Only a small percentage of the children experienced 4 or more FSAs/NLEs. With regard to cumulative stress (from FSAs and NLEs) and age, the percentage of children with no FSAs or NLEs decreases with age (which means that fewer and fewer children are shielded from adversities with increasing age), while the proportion of children with a higher number of stressors increases with age. Furthermore, there is no significant sex difference for cumulative stress from FSAs and NLEs (p=0.266 for FSA, p=0.688 for NLE).

**Table 2:** Prevalence of cumulative stress from FSAs and NLEs in pre- and primary-school children participating in the IDEFICS study (2009–2010): specifics for country groups, age groups and sex (FSAs: Familial and social adversities; NLEs: Negative life events)

		**Sum of familial and social adversities (FSAs)**						**Sum of negative life events (NLEs)**					
			**0**	**1**	**2**	**3**	**≥ 4**		**0**	**1**	**2**	**3**	**≥ 4**
**Country groups**	***N***	***Mean sum (SD)***	***Prevalence %***					***Mean sum (SD)***	***Prevalence %***				
North ^a^	1330	0.84 (0.989)	46.9	31.4	14.7	5.5	1.5	0.74 (0.901)	50.3	30.6	14.4	4.1	0.7
East ^b^	666	0.69 (0.858)	51.7	31.5	13.4	2.9	0.7	0.70 (0.941)	53.9	29.1	11.4	4.1	1.5
South ^c^	1660	0.91 (0.975)	41.7	34.9	16.3	5.4	1.7	0.35 (0.615)	71.0	23.7	4.4	0.9	0.1
West ^d^	981	0.80 (1.041)	51.1	28.2	12.9	5.8	1.9	0.62 (0.861)	57.4	27.6	10.9	3.4	0.7
**Age groups**	*N*												
4	258	0.67 (0.906)	55.4	28.3	11.2	3.9	1.2	0.53 (0.739)	60.1	28.3	10.1	1.6	0.0
5	582	0.72 (0.918)	52.1	30.6	11.9	4.3	1.2	0.47 (0.727)	64.8	25.9	7.7	1.0	0.5
6	736	0.79 (1.016)	51.1	28.9	13.2	4.8	2.0	0.52 (0.781)	62.4	26.6	8.4	2.2	0.4
7	605	0.72 (0.898)	51.2	30.9	13.2	3.8	0.9	0.47 (0.710)	63.8	26.9	7.8	1.3	0.2
8	735	0.76 (0.927)	48.6	32.9	13.7	3.5	1.2	0.52 (0.805)	63.0	26.1	7.2	3.1	0.5
9	1113	0.96 (1.016)	40.5	33.3	17.9	6.6	1.6	0.69 (0.910)	54.3	28.0	12.7	4.4	0.7
10	608	1.03 (1.041)	36.5	36.2	17.6	7.4	2.4	0.70 (0.919)	53.5	29.3	12.0	3.9	1.3
**Sex**	*N*												
Boys	2296	0.82 (0.952)	46.4	32.6	14.6	5.3	2.1	0.58 (0.830)	59.7	27.1	9.5	3.2	0.5
Girls	2341	0.84 (1.007)	46.8	31.4	14.8	5.0	2.0	0.57 (0.819)	59.7	27.4	9.8	2.4	0.7
**Total**	**4637**	**0.83 (0.980)**	**46.6**	**32.0**	**14.7**	**5.1**	**1.4**	**0.57 (0.825)**	**59.7**	**27.3**	**9.6**	**2.8**	**0.6**
		**(range 0–6)**						**(range 0–5)**					

^a^ Sweden - Estonia, ^b^ Hungary, ^c^ Italy-Cyprus-Spain, ^d^ Belgium-Germany.

### Associations and risk for adversities

Table 3 demonstrates that variables concerning socio-economic characteristics of the child’s life (being immigrant, family economic hardship, parental job loss, teenage pregnancy, low maternal education) are strongly interwoven with each other (e.g. children with low educated mothers are more likely to experience family economic hardship and children with family economic hardship are more likely to be immigrant) but are also associated with the family structure (parental divorce/separation, non-traditional family structure and only-children). A non-traditional family structure is not associated with family economic hardship (in contrast to parental divorce/separation), although single-parent families are 1.8 times more likely to experience economic adversity (data not shown, OR=1.80; 95% CI [1.26,2.59], p=0.001).

**Table 3:** Risk for co-occurring adversities in pre- and primary-school children participating in the IDEFICS study (2009–2010) (N=4637)

	**1**	**2**	**3**	**4**	**5**	**6**	**7**	**8**	**9**	**10**	**11**	**12**	**13**	**14**	**15**
**1. being immigrant** (not being immigrant as RC)															
**2. low maternal education** (no low maternal education as RC)	1.64*														
**3. family economic hardship** (no family economic hardship as RC)	1.64**	3.43*													
**4. non-traditional family structure** (traditional family structure as RC)	NS	1.69*	NS												
**5. only-children** (children with siblings as RC)	NS	1.34**	NS	2.89*											
**6. latchkey care** (no latchkey care as RC)	NS	0.36*	0.085*	1.63*	2.09*										
**7. bad family climate** (no bad family climate as RC)	NS	2.54*	2.74**	NS	NS	2.74**									
**8. teenage pregnancy** (no teenage pregnancy as RC)	2.01**	4.19*	NS	4.91*	2.31*	3.04*	3.43**								
**9. peer problems** (no peer problems as RC)	1.68**	1.46**	1.99*	1.53*	NS	NS	6.38*	2.07**							
**10. parental divorce/separation** (no parental divorce/separation as RC)	NS	1.39**	1.48**	92.5*	3.57*	2.65*	1.96**	5.99*	NS						
**11. addition of a new family member** (no addition of new family member as RC)	0.67**	NS	NS	4008*	NS	1.61**	NS	4.34*	NS	7.92*					
**12. parental job loss** (no parental job loss as RC)	NS	NS	5.18*	NS	1.34**	NS	2.22**	2.68*	1.4**	1.99*	1.47**				
**13. severe diseases/ accidents of the child** (no severe diseases/accidents as RC)	NS	NS	1.83**	NS	1.85*	NS	NS	NS	1.57**	1.53**	1.45**	1.93*			
**14. serious illness of a family member** (no serious illness family member as RC)	NS	NS	NS	NS	NS	NS	NS	NS	NS	NS	NS	2.7*	NS		
**15. major frustrations at school** (no major frustrations at school as RC)	NS	1.52**	NS	NS	NS	NS	NS	NS	2.86*	1.7*	1.81*	2.27*	2.48*	NS	

Only significant OR (univariate odds ratios) are presented. * significant at p<0.002 level; ** borderline significant (p>0.002 and p<0.05); NS not significant. This table presents the risk for co-occurrence of a specific adversity (first row) if another adversity is already present (first column) vice versa. ^a^RC= reference category.

Family climate also seems to be associated with socio-economic factors, with bad family climate being more likely with teenage pregnancy, low maternal education, family economic hardship, parental job loss, parental divorce/separation and latchkey care. Similarly, latchkey care is more likely to occur in children from mothers with teenage pregnancy, non-traditional family structure, parental divorce/separation and only-children. Latchkey care is however less likely to occur in children with low maternal education and family economic hardship.

## Discussion

To our knowledge, this study is the first to investigate both chronic and once-only adversities (i.e. FSAs and NLEs) in a large sample of European pre- and primary-school children, allowing us to study the influence of region, age and sex on the prevalence of adversities. Additionally, this study contributed to the knowledge of cumulative stress incidence and adversity-associations in a cross-national setting of young children. It should be noted that the prevalence and the types of reported FSAs or NLEs may vary according to the age of the children, ethnicity or culture, measurement approach and data collection methods and the period of assessment. The aim of the discussion-section below is thus to get an idea how our trends in childhood adversity (i.e. IDEFICS project) fit in the picture known from previous research.

### Prevalence of FSAs, NLEs and cumulative stress

Although a large percentage of the children was shielded from childhood stressors, a small group of children was exposed to multiple, accumulating adversities, which is in line with previous research [4,8]. Exposure to four or more FSAs or NLEs was reported for 1.4% and 0.6% of the children respectively, numbers which are however significantly lower than those reported by Furniss et al. [8].

While certain adversities occurred only rarely, others were very regular such as a non-traditional family structure and parental divorce/separation [4,8]. In general, this study indicated that one in five children does not live together with both biological parents. It can be assumed that with increasing age, this percentage increases. Schilling et al. [3] reported that by the age of young adulthood only one in two will live in an ‘intact two parent family’. Also in the IDEFICS project, similar trends were seen over time. In the baseline survey, 82.1% of the children lived in a two-parent-family [28], a proportion that had already decreased to 79% in the second survey period two years later, and which may further decrease during follow-up.

#### Influence of region

In accordance to EUROSTAT findings [26], this study indicated regional variations in living arrangements and family formations, with up to five-fold differences in the prevalence of parental divorce and non-traditional family structures. In general, children from northern countries seem to experience more parental divorce/separation and related difficulties (e.g. addition of a new family member, formation of stepparent families, latchkey care), while parents from southern countries reported more socio-economic adversities (e.g. being immigrant, family economic adversity). Remarkably, parental divorce/separation was less prevalent while single-parent families were more prevalent in southern countries compared to northern countries. This may indicate that in southern countries single-parent family structures are not necessarily related to divorce. Possibly, marrying rates may be lower and cohabitation may be more common in the southern survey centres. Fifteen-fold differences were observed for the prevalence of teenage pregnancies, although mean percentages were low. This may indicate that teenage pregnancies are becoming rare in most countries, which is in line with European findings of Robson and Berthoud [49]. Low maternal education prevalence largely varies between survey centres (i.e. high for Italy and Germany, while being low for the other countries) and was previously described by Ahrens et al. [28] as a possible selection effect at baseline, more specifically as an underrepresentation of low-income groups in some countries at baseline. Also the large difference in immigrant prevalence between Cyprus and Germany (high) compared to the Belgian cohort (low) has been discussed by Ahrens et al. in the context of historical aspects [28].

Although description of regional variations in this study aimed to be strictly exploratory, cultural, religious and welfare typologies should be considered in interpreting results. Cultural and religious characteristics such as the attitude towards contraception, marriage and divorce, or tri-generational families may affect the observed differences in family formation patterns (e.g. less prevalent divorce in the more Catholic southern countries). Also, differences in perception of ‘serious’ illnesses, ‘major’ frustrations and ‘bad’ family climates due to culture, may have influenced distinct prevalence percentages for some of the studied adversities. Last, the heterogeneity of societal and policy regimes within the studied countries should be considered in interpreting results on socio-economic welfare, educational chances etc.

#### Influence of age

The risk for childhood adversity generally increased with age. This did however not apply for some variables which were more constant over time (e.g. family economic hardship, bad family climates and being immigrant) and can therefore be considered ‘chronic’, persistent adversities [4]. Latchkey care increased by 12.7% over the age groups, suggesting that particularly children of the last years of primary school are more often left alone (after school).

#### Influence of sex

In the literature it has been indicated, although sometimes inconsistently, that sex differences may occur in the types of events experienced, possibly resulting from sex differences in social roles [25]. In this study we could however not demonstrate such sex differences for the studied FSAs and NLEs, except for the occurrence of severe diseases/accidents of the child and peer problems (in the age group of 9 year olds) which were more frequent in boys (borderline significant). Our findings can be explained by significant differences in peer relationships in boys and girls as shown by Rose et al.: girls have been shown to engage in more prosocial interactions with higher self-disclosure in friendships and to empathize with others, while boys have been shown to more frequently engage in organized play (e.g. sports, competitive games, rough-and-tumble play), to emphasize the importance of self-interest and dominance within their peer group and to encounter more peer stress in the form of overt physical or verbal victimization [60]. Our findings (i.e. more frequent diseases/accidents and peer problems in boys) thus fit within this described context.

### Associations and risk for adversities

The present findings showed that negative life events and chronic adversities tend to cluster or co-occur (although no statements on direction or causality can be made), i.e. children exposed to a certain NLE or FSA are likely to also be exposed to other socio-economic or familial adversities, all together shaping the living conditions of the child and possibly resulting in cumulative childhood stress. In the context of the indicated connection between socio-economic and familial variables (Table 3), teenage pregnancy was (similar to findings of Robson and Berthoud [49]) more likely to co-occur with less preferable economic and family situations for the child. Also in line with previous research [4], we identified a relationship between parental divorce, single-parent families and family economic adversity. Bad family climates were more likely to occur in families with divorced or separated parents, but not in non-traditional family structures, which may postulate the impact of divorce itself on family tensions and on the parental ability and opportunities to effectively interact with their children [41,61]. Furthermore, bad family climates were more likely to take place in families with low educated mothers, which may point to a relationship between the mother’s education and the way of interacting with the child and the parent–child relationship [62]. Children with peer problems were 6 times more likely to experience bad family climates (and vice versa), suggesting an interrelatedness between social and familial relationships. Despite limited financial resources, families with economic hardship and low educated mothers showed less latchkey care, which resembles previous research and may be explained by a more frequent presence of the mother at home due to less frequently being fully-employed [37,38]. Latchkey care was however more likely in non-traditional family structures speculating that parents from these family structures may receive less help from e.g. a life partner in after-school child-care. Two more remarks relate to only-children. The finding that only-children are more likely to experience latchkey care may be quite obvious since children that are left alone with older siblings are strictly speaking not ‘left alone’ and may thus be less reported.

### Strengths and limitations

The strength of this study is its large, international sample comprising 8 European countries from north, east, south and west, allowing us to study childhood adversity in a larger context than has previously been done and allowing insightful comparisons across different nations in children younger than 12 years old, by investigating both once-only and more chronic situations. In addition, all survey centres were studied at the same time using the same, standardized protocol. Nevertheless, some weaknesses may lay in some specific methodological aspects: 1) the dichotomous nature of the variables may not consider the complexity of certain issues (e.g. immigration, family structure), 2) only a limited number of NLEs and FSAs were assessed, which were exclusively parent-reported and did not take into account children’s perspectives; also the fact that only biological-, adoptive-, or stepparent reported data on maternal education, family economic hardship and family climate was included, could have excluded the most affected children, 3) measures of NLEs may be underestimated because of their retrospective nature (possible recall bias) and the lack of differentiation between ‘no occurrence of the event’ or ‘missing information’ in the NLE questionnaire (although, it is quite likely that serious events such as deaths etc. are reported quite accurate, while other events such as e.g. major frustrations at school are difficult to report by parents and may as well be overestimated), 4) a selection or non-participation bias related to education or income-level, as well as a response bias cannot be ruled out and may thus have influenced prevalence results (since respondents might differ in characteristics from non-respondents and since respondents may have the tendency to give a “morally right” answer) [28], and to end 5) it is noteworthy that the selected communities are not necessarily representative for each country. Comparisons between countries should therefore be made with caution.

## Conclusion

Next to showing variations in the prevalence of childhood adversities across regions, age groups and sex, this study demonstrated the co-occurrence and connection between socio-economic adversities and family characteristics, which all together shape the living conditions of the child and which may possibly result in cumulative childhood stress in children younger than 12 years old. Even though family formation change and disadvantage in the early family or social environment may not harm all children equally, they should not be considered risk-free living conditions given their widespread appearance, consequences on family life and long-term health risk (although it should be noted that some family changes may be protective for the children by removing them from conflicted or violent households). The importance of future recording/monitoring potential childhood adversities in pre- and primary-school children lies within the further elucidation of the mental and physical health consequences of childhood adversities and the possibility for short- and long-term prevention of adverse health effects.

## Competing interests

There are no competing interests

## Authors’ contributions

All authors have made a substantial contribution to this manuscript based on the three conditions: study design, manuscript editing and final approval. All authors were responsible for the practical organization and data collection in the different IDEFICS’ survey centers. BV was the major responsible person for manuscript drafting. BV, IH, KB and IDB were involved in statistical analyses. All authors contributed to the critical evaluation of the paper. The manuscript was read and approved by all authors.
